# Epigenetic Dynamics of the Infant Immune System Reveals a Tumor Necrosis Factor Superfamily Signature in Early Human Life

**DOI:** 10.3390/epigenomes4030012

**Published:** 2020-07-04

**Authors:** Maria J. Gutierrez, Gustavo Nino, Xiumei Hong, Xiaobin Wang

**Affiliations:** 1 Division of Pediatric Allergy, Immunology and Rheumatology, Department of Pediatrics, Johns Hopkins University, Baltimore, MD 21287, USA; 2 Division of Pediatric Pulmonary and Sleep Medicine, Children’s National Medical Center, George Washington University, Washington, DC 20010, USA; 3 Center for Genetic Medicine, Children’s National Medical Center, Washington, DC 20010, USA; 4 Center on the Early Life Origins of Disease, Department of Population, Family and Reproductive Health, Johns Hopkins University Bloomberg School of Public Health, Baltimore, MD 21205, USA; 5 Division of General Pediatrics & Adolescent Medicine, Department of Pediatrics, Johns Hopkins University School of Medicine, Baltimore, MD 21287, USA

**Keywords:** DNA methylation, immunology, early life, tumor necrosis factor cytokine superfamily, antibody production

## Abstract

DNA methylation (DNAm) is an essential mechanism governing normal development in humans. Although most DNAm patterns in blood cells are established *in utero*, the genes associated with immune function undergo postnatal DNAm modifications, and the characterization of this subset of genes is incomplete. Accordingly, we used available longitudinal DNAm datasets from a large birth cohort in the U.S. to further identify postnatal DNAm variation in peripheral leukocytes from 105 children (*n* = 105) between birth and the first two years of life, as determined by postnatal changes in β values (with the percentage of methylation ranging from 0 to 1.0 at individual CpG sites). Our study is an extension of a previous analysis performed by our group and identified that: (1) as previously described, DNAm patterns at most CpG sites were established before birth and only a small group of genes underwent DNAm modifications postnatally, (2) this subset includes multiple immune genes critical for lymphocyte development, and (3) several members of the tumor necrosis factor receptor and cytokine superfamilies with essential roles in immune cell activation, survival, and lymphoid tissue development were among those with a larger postnatal variation. This study describes the precise epigenetic DNA methylation marks in important immune genes that change postnatally and raises relevant questions about the role of DNAm during postnatal immune development in early childhood.

## 1. Introduction

DNA methylation (DNAm) is an epigenetic mechanism essential for normal development [1,2]. Changes in global or regional DNAm regulate the chromatin structure and important developmental processes such as X chromosome inactivation, allelic imprinting and long-term gene silencing [1–3]. DNAm patterns are also well correlated with chronological age in humans and are tissue-specific [4–6]. In blood cells, the individual DNAm patterns during early life are largely established prenatally, and only a small group of genes undergo DNAm changes in the first years of life [7,8].

This subset is enriched for genes associated with the immune system [7–9], which rapidly mature in the early postnatal years [10]. However, the characterization of this particular group of genes is incomplete. Accordingly, we aimed to expand the study of longitudinal DNAm changes in genes associated with immune function during early life using data previously generated by our group [7].

## 2. Results and Discussion

**Figure 1.**
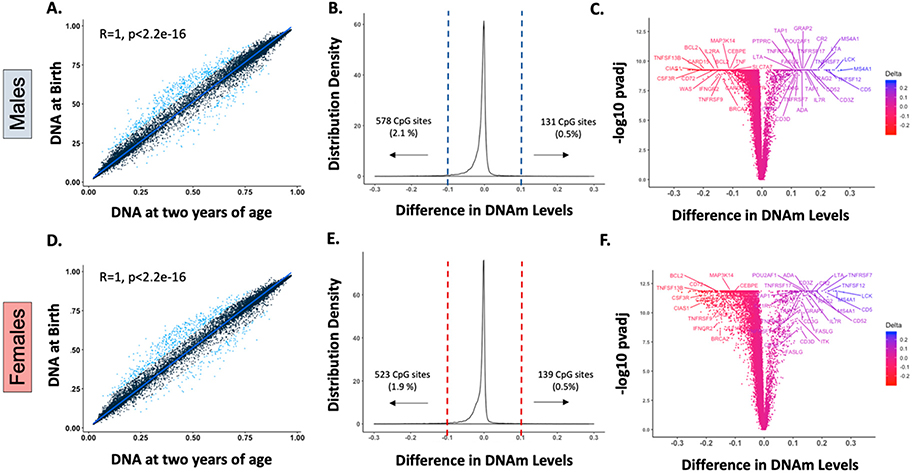
Only a small number of genes undergo postnatal DNA methylation (DNAm) modifications, and multiple genes associated with immune cell development are identified in this group. (**A**,**D**): Spearman correlation of intra-individual birth and postnatal mean DNAm levels (β values) demonstrated a stable postnatal pattern (*r* > 0.99, *p* < 0.000) for most of the genes assessed in males (**A**) and females (**D**). Blue dots represent probes with a significant postnatal mean DNAm variation (changes in β value > 10%, *p* values < 0.05). (**B**,**E**): Density plots of the intra-individual differences in β values at 27,549 CpG sites in males and females. The difference between postnatal and fetal DNAm levels is less than 10% for most of the CpG sites assessed, and only a small subset exhibited significant methylation (~0.5% of CpG sites) or demethylation (~2% CpG sites) postnatally in both sexes. (**C**,**F**): Immune genes with statistically significant postnatal DNAm differences are labeled in males (**C**) and females (**F**). Genes annotated to CpG sites with significant early life DNAm changes included multiple lymphocyte development genes such as those that encode for DNA-breakage enzymes (e.g., *RAG2*, *ADA*), lymphocyte activation/costimulation factors (e.g., *IL7R*, *IL21R*, CD3 chains), and T-cell and B-cell receptors signaling enzymes (e.g., *LCK*, *ITK*). Several genes encoding tumor necrosis factor (TNF) superfamily receptors (e.g., *TNFRSF4*, *TNFRSF7*, *TNFRSF9*, *TNFRSF17*) and cytokines (e.g., *TNF*, *FASLG*, *LTA*, *TNFSF12*, *TNFSF13B)* were among those with larger postnatal variation.

As previously described [7], our analysis of DNAm in whole blood leukocytes showed that intra-individual birth and postnatal mean β values, had a stable pattern (*r* > 0.99, *p* < 0.000) in both sexes after birth, confirming that DNAm is predominantly established in utero (Figure 1A,D). Nonetheless, there is a small subset of genes that exhibited longitudinal changes in the first two years of life, defined by statistical significance (*p* value < 0.05) and magnitude (>10% change in the mean β-value (Tables S1 and S2)). In our analyses, 578 (2.1%) and 131 (0.5%) CpG sites out of 27,549 probes underwent postnatal demethylation or methylation, respectively, in males (Figure 1B). In females, 523 (1.9%) CpG sites were demethylated, and 139 (0.5%) were methylated after birth (Figure 1E). Interestingly, there were 125 methylated and 506 demethylated CpG sites common to males and females, with changes of similar magnitudes, suggesting that these modifications are conserved between sexes. These CpG sites with postnatal modifications were mapped to less than 5% of the 14,474 genes annotated in the array, and postnatal DNAm differences ranged between −0.292 (~29% demethylation) and 0.272 (~27% methylation (Figure 1B,E)).

Interestingly, these early life DNAm modifications included multiple genes that are essential for lymphocyte development (Figure 1C,F) [11,12] such as those encoding for DNA-breakage enzymes (e.g., *RAG2*, *ADA*), lymphocyte activation/costimulation factors (e.g., *IL7R*, *IL21R*, CD3 chains), and TCR/BCR signaling enzymes (e.g., *LCK*, *ITK*). Of note, several receptors and cytokines of the TNF superfamily were among those genes that exhibited more substantial postnatal DNAm variation. The TNF superfamily (TNFSF) is a group of 19 structurally related cytokines, that, through their cognate receptors (29 members of the TNF receptor superfamily, TNFRSF), induce important cellular responses [13–15]. In the immune system, the TNFSF and their receptors developed in parallel with adaptive immune responses in vertebrates [16] and have critical roles in immune homeostasis, including cell differentiation, development of lymphoid tissues, lymphocyte costimulation, and cell survival and death [13,17].

**Figure 2.**
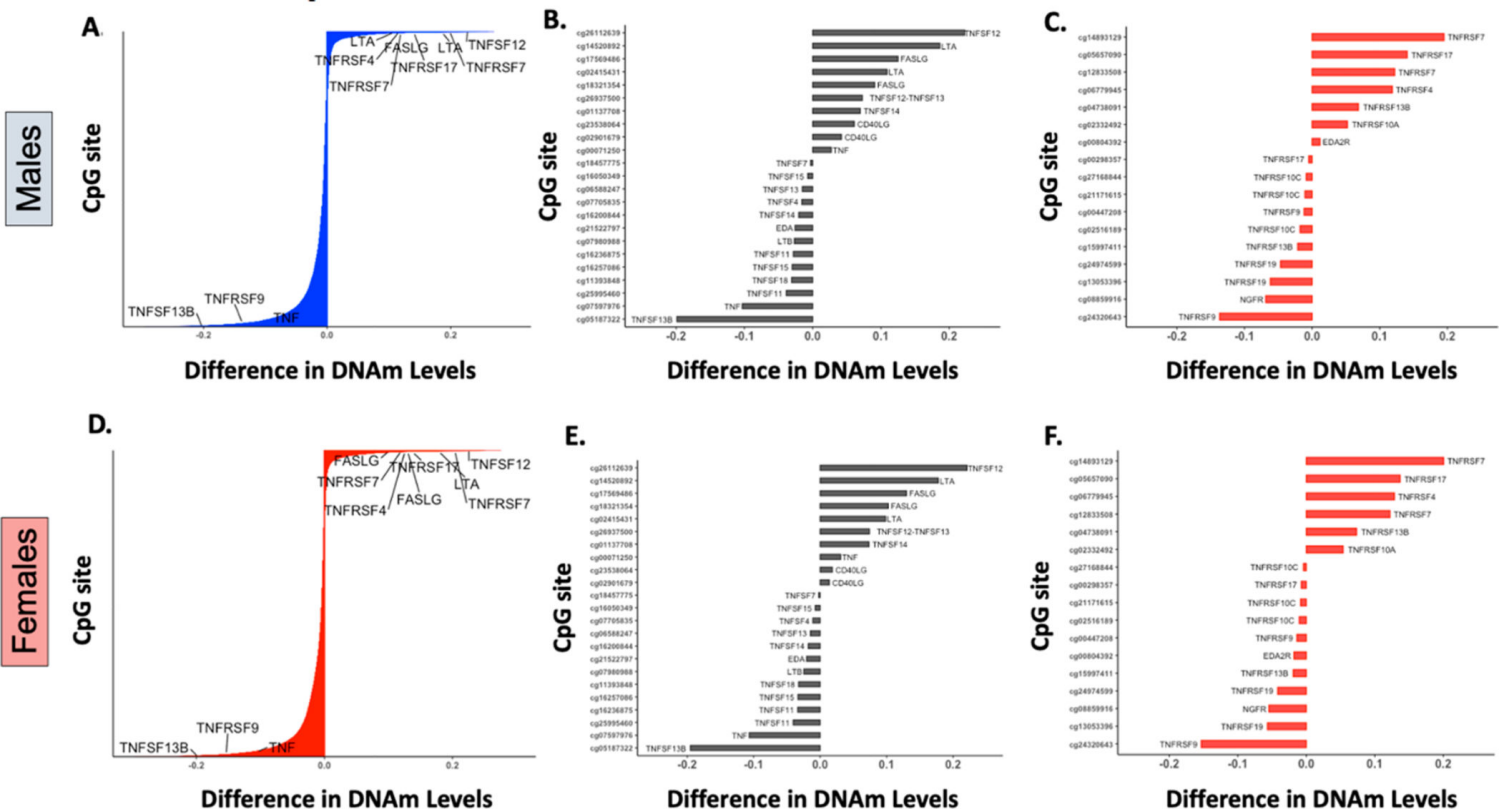
DNA methylation changes in members of the TNF cytokine and receptor superfamilies. (**A**,**D**): Barplots displaying the differences between fetal and postnatal DNAm levels at individual CpG sites in males (**A**) and females (**D**). The Y-axis represents 27,549 individual CpG sites in the array (labels are omitted). Labeled probes are annotated showing the five receptors and four cytokines involved in the TNF superfamily cytokine signaling that underwent significant DNAm changes (*p* value < 0.05) greater than 10% after birth.(**B**,**C**,**E**,**F**): Barplots representing CpG sites mapped to TNF superfamily cytokines (**B**,**E**) or to TNF superfamily receptors (**C**,**F**), with statistically significant postnatal differences (*p* value < 0.05) in mean DNAm levels (β values) for males and females.

Genes encoding four TNFRSF members and five TNFSF cytokines underwent statistically significant DNAm changes greater than 10% in β values after birth (Figure 2A,D). As previously described [7], receptors that regulate the maintenance of long-term T-cell and humoral immunity, namely *TNFRSF7* (encoding CD27) [13], required for T-cell survival and antibody production, and *TNFRSF17* (protein encoded B-Cell Maturation Antigen or BCMA), essential for plasma cell survival [18], were the top two postnatally methylated genes among the 29 known members of the receptor superfamily (Figure 2C,F) [13,17]. Additional receptors that underwent significant postnatal DNAm changes (>10% change in β value) included the *TNFRSF4* gene (encoding OX40), important for T- and B-costimulation, T-cell survival, and antibody production which was methylated, and the *TNFRSF9* gene (encoding 4–1BB), an inducible stimulatory receptor expressed on T-cells and innate lymphoid cells that was demethylated [13]. In addition to receptor genes, the ligands *TNFSF12* (protein encoded TNF-related weak inducer of apoptosis, TWEAK), *LTA* (encoding lymphotoxin α, LTα), and *FASLG* (encoding Fas ligand, FasL), which have dual co-stimulatory and pro-apoptotic properties on immune cells [13,17], were also methylated after birth (Figure 2B,E). In contrast, *TNFSF13B* (protein encoded B-cell activation factor, BAFF), one of the main ligands to all the B-cell lineage survival receptors [19] and *TNF* (encoding tumor necrosis factor, TNF) were the top demethylated genes among those encoding TNF family cytokines with a 20% and 10% decrease in β values after birth (Figure 2B,E).

Of note, although they show a less pronounced postnatal DNAm variation (5–10% increase or decrease in β values), two additional TNFSF and four TNFRSF genes underwent significant DNAm changes during the first two years of life. The receptor genes *TNFRSF13B* (protein encoded transmembrane activator and calcium-modulating cyclophilin ligand interactor, TACI), key for B-cell survival, and *TNFRSF10A* (encoding Death Receptor 4, DR4), as well as the cytokine genes *TNFSF12–13* (encoding TWE-PRIL, a fusion protein of TWEAK and APRIL) and *TNFSF14* (encoding LIGHT) were methylated after birth. In contrast, genes encoding TNF receptor superfamily member 19 (TNFRSF19/TROY) and the nerve growth factor receptor (NGFR) were postnatally demethylated (Figure 2B,C,E,F). Most postnatal DNAm changes, annotated as TNF superfamily cytokines or receptor genes in our study, were located in non-CpG islands, mainly upstream of the transcription start site (TSS1500) and at gene bodies of some genes (e.g., *TNFRSF7, TNFSF12, TNFSF13B,* and *LTA*), or in the first exon or 5′ UTR regions of others (e.g., *TNFRSF17*, *LTA, TNF* and *FASLG*).

Together, our findings raise questions as to why genes that critically govern immune cell development and function undergo postnatal DNAm modifications. The observed changes in DNAm may correspond to shifts in immune cell populations known to occur in the first years of life [20]. It is also possible that DNAm patterns are modified in individual cell populations as the result of cell maturational changes (e.g., the emergence of memory T- and B-cells and antibody secreting cells) during the first postnatal years. More in-depth studies of the epigenetic dynamics of the infantile immune system are needed to elucidate cell-specific DNAm changes that occur with age in young children. It would also be relevant to examine whether the observed changes vary across children (suggesting influences from individual non-inheritable factors) or, on the contrary, correspond to stereotypical changes. Further characterization of age-related epigenetic variation in genes governing immune cell development, including the TNF superfamilies of cytokines and receptors, during early life, is important for gaining additional insights into the mechanisms of immune development during this critical period.

A limitation of our study is that only a limited number of CpG (27,578 probes) were assessed, which may not reveal the full spectrum of DNAm changes affecting the TNF superfamily signaling and immune development pathways. Second, the presence of single nucleotide polymorphisms (SNPs) near CpG sites may affect the accuracy of DNA methylation measurements. Additionally, as DNAm profiles were obtained from a mixed cell population in whole blood samples, it was not possible to identify cell-specific DNAm variations, and cell heterogeneity in the whole blood samples may have confounded our findings. Finally, as our study included only black children, it remains to be determined if our results can be replicated in other race/ethnicity children.

Nonetheless, our study provides insights into the epigenetic profile of key immune development pathways in early life by using a longitudinal analysis of human infant blood samples. Our findings additionally generate relevant questions about how the observed DNAm modifications of members of the TNF cytokines are associated with early postnatal immune development. Further characterization of the postnatal DNAm modifications in immune genes and their biological significance in the infantile human immune system is warranted. Considering that the epigenome is modifiable and susceptible to non-inheritable stimuli (e.g., environmental, nutritional, biochemical), elucidating whether, why, how and when postnatal DNAm modifications in the TNF cytokine superfamily signaling and other essential immune development genes occur would advance our understanding of epigenetic control of immunity during early postnatal years.

## 3. Materials and Methods

To study postnatal DNAm profiles, we used longitudinal genome-wide DNAm datasets previously generated from the Boston Birth Cohort (BBC) [7]. Children in the BBC are enrolled under protocols approved by the Institutional Review Board (IRB) at Boston Medical Center. Genome-wide DNAm data from 105 singleton (*n* = 105), full-term black children (59 males and 46 females) obtained from peripheral leukocytes at birth (cord blood) and within the first two years of life (venous blood) were analyzed. Original DNAm datasets were generated using the Illumina Human Methylation27 BeadChip and a description of the quality control and epigenetic mapping used were previously published [7]. The DNAm level was measured as the percentage of methylation, ranging from 0 to 1.0 (β value), at individual CpG sites. DNAm differences between birth and postnatal measurements were evaluated using the Wilcoxon Signed-rank test, and the Benjamini and Hochberg false discovery rate (FDR) was used to adjust the *p* values for multiple testing [7]. For the present analysis, datasets with average birth and postnatal β values were available for 27,549 probes annotated to 14,474 genes in males and females [7]. The data analysis was conducted using STATA 16 (StataCorp, College Station, TX, USA, 2019) and R studio (R Core Team, 2019). Spearman’s correlation was used to evaluate the relationship between the intra-individual birth and postnatal mean β values. Postnatal DNAm changes were calculated as the difference between the average birth and postnatal β values for each probe. Significant postnatal DNAm changes were defined as changes greater than 0.1 (10%) in β values at individual CpG sites with multiple comparisons corrected *p* values (FDR) less than 0.05. For identification of DNAm variation in immune genes, we examined DNAm patterns in 524 genes knowingly important for lymphocyte development [11,13] or established causes of human inborn errors of immunity [12].

## Supplementary Material

Supplementary
